# Relationship Between Stroke Knowledge, Health Information Literacy, and Health Self- Management Among Patients with Stroke: Multicenter Cross-Sectional Study

**DOI:** 10.2196/63956

**Published:** 2025-06-23

**Authors:** Mengxue Zeng, Yanhua Liu, Ying He, Wenxia Huang

**Affiliations:** 1 Health Management Center, General Practice Medical Center, West China Hospital, Sichuan University, 37 Guoxue Lane, Wuhou District, Chengdu, 610041, China, 86 13980589954; 2 West China School of Nursing, Sichuan University, Chengdu, China

**Keywords:** stroke, health information literacy, self-management, stroke knowledge, mediating effect

## Abstract

**Background:**

The World Health Organization highlights the essential role of effective self-management in the prevention and control of chronic diseases, noting that proper self-management can significantly slow disease progression. Stroke, which ranks as the third leading cause of death worldwide, is often accompanied by inadequate self-management among patients. While health information literacy (HIL) has been shown to influence self-management in conditions such as diabetes and hypertension, its role as a mediating factor linking disease perception and health behavior in patients with stroke remains insufficiently explored. Addressing this research gap is vital for developing targeted interventions.

**Objective:**

The aim of this study was to investigate the current status of HIL, stroke knowledge, and self-management abilities among patients with stroke in Southwest China. Additionally, the study analyzed the relationships among these three factors and their mechanisms of action, providing evidence to inform the development of effective health education strategies for enhancing self-management in patients with stroke.

**Methods:**

A multicenter cross-sectional design was employed, enrolling 514 patients with stroke from the neurology departments of three tertiary general hospitals in Chengdu between September 2022 and March 2023. Data collection used a standardized set of scales: the health information literacy questionnaire for stroke assessed HIL, the stroke prevention questionnaire evaluated knowledge levels, and the stroke self-management assessment scale measured self-management abilities. Regression analysis and bootstrap mediation effect testing were conducted using SPSS software (version 26.0).

**Results:**

Patients with stroke had a mean (SD) score of 17.61 (6.46) for stroke knowledge, 61.17 (13.58) for HIL, and 158.70 (19.07) for self-management skills. Correlation analysis indicated a positive correlation of stroke knowledge with both self-management (*r*=0.668; *P*<.001) and HIL (*r*=0.138; *P*<.001). The mediation test showed a significant mediating effect of HIL between stroke knowledge and self-management (*β*=0.543; 95% CI: 0.431‐0.663), with an effect share of 82.77%.

**Conclusion:**

There is a correlation between HIL and self-management; the higher the HIL, the better is the self-management behavior. Furthermore, HIL partially mediates the effect of stroke knowledge on self-management.

## Introduction

Stroke is the leading cause of adult mortality worldwide, particularly among Chinese adults, with a rapidly increasing incidence, poor prognosis, mortality, and varying degrees of disability among survivors. This imposes a significant economic burden on patients’ families and society [1,2]. Inadequate health information about stroke leads to delayed consultation and suboptimal prevention and control due to the lack of timely disease-related relief [3]. Therefore, increasing the awareness of patients with stroke has a positive impact on the prevention and management of stroke. In particular for the population with poststroke dysfunction, low health information literacy (HIL) affects treatment and recovery and significantly reduces their quality of life. Research has shown that improving HIL in patients with stroke is crucial to promoting overall well-being, as it improves public understanding of health and disease and positively influences patients’ self-management practices [4,5]. Effective self-management can motivate patients to improve unhealthy lifestyles, increase their ability to monitor their disease, improve adherence to treatment, and consequently improve their quality of life [6]. HIL combines the concepts of health literacy and information literacy; it focuses on an individual’s ability to obtain, understand, and use health information and services, and to make appropriate judgments and decisions to maintain and improve their health [7,8]. Existing studies have primarily investigated the current status of HIL in patients with stroke, with less investigation into the possible mediating effect of HIL on knowledge levels and self-management skills [9-11]. Therefore, the aims of this study were to analyze the relationship between stroke knowledge, HIL, and self-management in patients with stroke, to examine the mediating effect of HIL, to provide new ideas for interventions to improve patients’ self-management ability, and to target the implementation of health education for patients with stroke.

## Methods

### Setting and Samples

This was an observational, multicentere, cross-sectional study, which included hospitalized patients with stroke from September 2022 to March 2023 in the emergency and critical care center of tertiary hospitals in three stroke centers: West China Hospital of Sichuan University, West China Tianfu Hospital of Sichuan University, and West China Shangjin Hospital of Sichuan University. The patient recruitment process of this study strictly adhered to the ethical review requirements as follows: physicians initially screened eligible patients with stroke according to the inclusion and exclusion criteria of the study, and after obtaining preliminary consent from the patients, the research team conducted extensive face-to-face communication with the patients. The survey consisted of three main questionnaires, and each participant took approximately 15‐20 minutes to complete all the questionnaires; all the participants’ questionnaires were collected at once. Quality control measures were implemented during the data collection process, with researchers receiving uniform language training and participants being interviewed face-to-face and asked to complete the questionnaires independently. For patients who were unable to complete the questionnaire, the investigators asked the items neutrally and objectively to ensure the accuracy of the questionnaire.

The inclusion criteria were (1) stroke diagnosis based on computed tomography or magnetic resonance imaging results, (2) mental and physical fitness to participate, and (3) willingness to participate and cooperate. The exclusion criteria were (1) severe complications and loss of self-management, (2) associated with psychiatric disease or intellectual disability, (3) unwillingness to participate in the survey, or (4) participation in other research projects at the same time.

### Variables and Instruments

#### Sociodemographic and Clinical Characteristics

Based on the characteristics of stroke and relevant studies [12,13], the general information form included socio-demographic and disease-related information. The socio-demographic information included age, gender, residence, marriage, living conditions (primary caregiver), education level, occupation, history of tobacco and alcohol consumption, and type of health insurance. Disease-related data included age at onset and first symptoms. All variables above were categorical, and complete data were obtained.

#### The Stroke Prevention Questionnaire

The Stroke Prevention Questionnairewas developed by Zhang [14] to assess stroke knowledge of patients with stroke and contains 36 items with 8 dimensions (Cronbach α=.791), including lifestyle, diet, exercise, knowledge of risk factors, medication, blood pressure monitoring, knowledge of stroke signs and stroke management. Each item was scored on a scale of 0 and 1, where 0 indicated “wrong answer” or “don’t know” and 1 indicated a “correct answer.” The total score ranged from 0 to 36; the higher the score, the better was the stroke outcome.

#### The HIL Questionnaire for Stroke

The HIL questionnairewas a specific HIL assessment tool developed by Yao [15] and included 5 dimensions of health information with 21 items, such as health information awareness (4 items), acquisition (4 items), comprehension (4 items), evaluation (6 items), and application (3 items). The scale followed a Likert-5 scale, with each item having a minimum score of 1 and a maximum score of 5. The total score ranged from 21 to 105, with higher scores indicating higher levels of HIL. The instrument showed good reliability. The overall Cronbach α of the scale was 0.915, and the Cronbach α of each dimension ranged from 0.745 to 0.878, indicating a better model fit index and high structural validity.

#### The Stroke Self-Management Assessment Scale

The stroke self-management assessment scale was developed by Wang [16] and consisted of 50 items in 7 dimensions, including disease management, medication management, diet management, daily life management, emotional management, social and interpersonal management, and rehabilitation management. The options were mostly “no,” “occasionally,” “sometimes,” “often,” and “always,” with total scale scores ranging from 50 to 255. Higher scores indicated better self-management. The scale had a Cronbach α of 0.835, content validity of 0.95, and structural validity of 0.594‐0.771. The scores of the different dimensions were compared using the standard score index. The formula for the standard score index = (the actual score of this dimension/the highest score of the dimension) × 100%. The standard score index of <60% indicated poor self-management, 60%‐80% indicated moderate self-management, and >80% indicated good self-management.

### Data Analysis

SPSS software (version 26.0; IBM Corp) was used for data analysis, and Microsoft Excel spreadsheets were used for data entry. Quantitative data were expressed as mean (standard deviation), and comparisons between groups were made using *t* tests or one-way analysis of variance. Categorical data were expressed as proportions (%), and comparisons between groups were made using the *χ*^2^ test. Regression analysis was performed to determine the relationship between HIL, stroke knowledge, and self-management. Regression analysis was also used to test whether HIL in patients with stroke mediated the relationship between stroke knowledge and self-management based on relevant analysis results. A *P* value <.05 was considered statistically significant.

### Ethical Considerations

Prior to data collection, the study was approved by the Ethics Committee on Biomedical Research, West China Hospital, Sichuan University (2023 Review (688)). All participants provided consent to participate in the study on a voluntary basis, and the data were anonymized. No benefits or compensation were offered or given to any participant.

## Results

### Characteristics of Participants

**Figure 1.**
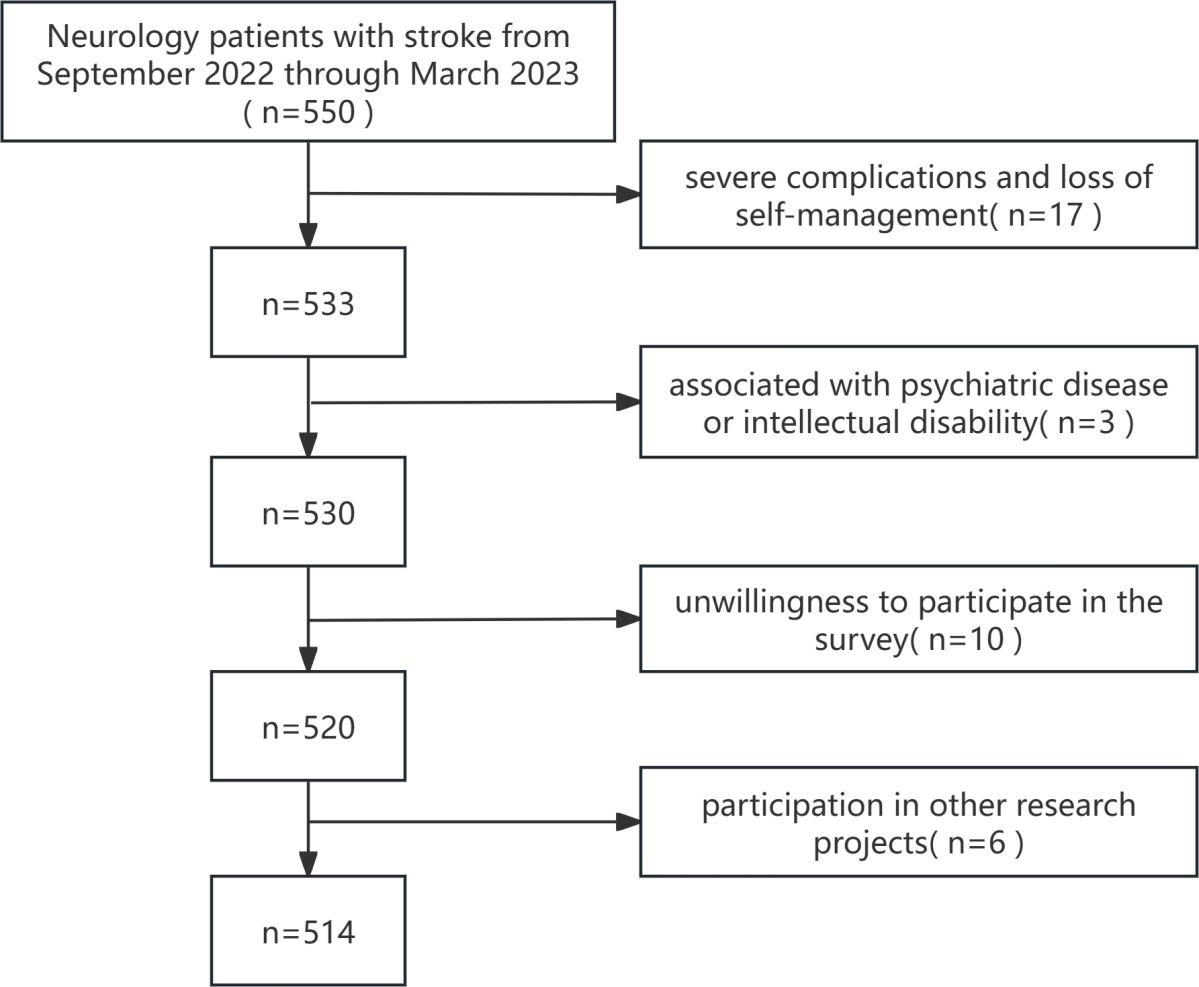
Flowchart for including and excluding study participants.

A total of 550 stroke survivors were recruited and 514 valid questionnaires were recovered, with a valid recovery rate of 93.45%. The flowchart showing the inclusion and exclusion of patients is shown in Figure 1.

The general characteristics of the study participants are given in Table 1. Of the 514 participants, 338 (65.8%) were male and had a mean (SD) self-management ability score of 157.05 (18.78) points. Of the 514 participants, 465 (90.4%) of the participants were aged 50‐85 years, among whom the minimum age of first stroke was 20 years. Furthermore, the distribution of residence locations revealed that the majority of the participants, 331/514 (64.4%), resided in urban areas, with a substantial proportion having completed middle school as their highest level of education (185/514, 36%). The initial incidence of stroke was predominantly observed among individuals aged 60 years and older.

**Table 1.** General information of patient participants.

Variable	n (%）	Self-management ability score, mean (SD)	*t* test or *F* test *(df)*	*P* value
Gender			*t*=2.754 (512)	.006
Male	338 (65.8)	157.05 (18.78)		
Female	176 (34.2)	161.90 (19.26)		
Age years			*t*=-2.461 (512)	.014
<50	49 (9.5)	152.37 (18.45)		
50-85	465 (90.5)	159.38 (19.03)		
Residence			*t*=6.328 (398.95)	<.001
Urban	331 (64.4)	162.46 (18.87)		
Rural	183 (35.6)	151.95 (17.55)		
Education			*F*=10.981 (3, 510)	<.001
Elementary school	154 (30)	153.10 (18.07)		
Middle school	185 (36)	157.73 (17.47)		
High school or secondary school	81 (15.8)	164.36 (21.20)		
College and above	94 (18.3)	164.99 (18.89)		
Previous occupation			*F*=26.771 (3, 510)	<.001
Manual workers	216 (42)	154.87 (18.23)		
Commercial or service workers	153 (29.8)	153.70 (15.72)		
Professional and technical workers	143 (27.8)	169.48 (19.16)		
Freelance or unemployed	2 (0.4)	188.50 (3.54)		
Marriage			*F*=2.42 (3, 510)	.065
Unmarried	14 (2.7)	154.36 (19.38)		
Married	67 (13)	164.31 (21.17)		
Divorces	7 (1.4)	156.14 (16.93)		
Widowed	426 (82.9)	158.02 (18.65)		
Living conditions			*F*=1.239 (2, 511)	.290
Alone	38 (7.4)	160.95 (23.43)		
Cohabitation with family	471 (91.6)	158.66 (18.56)		
Nursing home	5 (1)	146.80 (29.55)		
Smoking status			*t*=4.036 (512)	<.001
No	243 (47.3)	162.25 (18.36)		
Yes	271 (52.7)	155.55 (19.17)		
Alcohol consumption			*t*=1.244 (489.50)	.214
No	260 (50.6)	159.75 (17.16)		
Yes	254 (49.4)	157.65 (20.83)		
Age at first incidence of stroke (years)			*F*=5.516 (3, 510)	.001
<40	15 (2.9)	146.73 (19.59)		
40-50	39 (7.6)	156.69 (16.50)		
50-60	151 (29.4)	155.38 (19.13)		
≥60	309 (60.1)	161.18 (18.91)		

### Stroke Knowledge, HIL, and Self-Management Scores

The mean (SD) estimated stroke knowledge score of 514 patients with stroke was 17.61 (6.46) points, the mean (SD) total score on the HIL scale was 61.17 (13.58) points, and the mean (SD) total score on the stroke self-management assessment scale was 158.70 (19.07) points. The scores for each dimension are shown in Table 2.

**Table 2.** The scores of stroke knowledge, health information literacy (HIL), and self-management (*x±s*).

Variable and dimension	Dimension score, mean (SD)	Total score, mean (SD)
Stroke knowledge^a^		17.61 (6.46)
Lifestyle	4.14 (2.00)	
Exercise	1.74 (1.21)	
Diet	2.64 (1.30)	
Risk factors	1.76 (1.26)	
Medication	2.76 (1.14)	
Blood pressure monitoring	1.33 (0.70)	
Stroke signs	2.69 (1.45)	
Management	0.54 (0.75)	
HIL^b^		61.17 (13.58)
Awareness	12.76 (3.30)	
Acquisition	7.92 (2.95)	
Comprehension	10.64 (3.52)	
Evaluation	16.44 (4.79)	
Application	13.42 (1.94)	
Self-management^c^		158.70 (19.07)
Disease	20.54 (8.24)	
Medication	20.20 (2.97)	
Diet	30.53 (4.67)	
Daily life	31.61 (3.48)	
Emotion	18.98 (4.10)	
Social and interpersonal	19.98 (2.97)	
Rehabilitation	16.89 (5.63)	

a Stroke knowledge: actual score ranged from 4 to 35.

b HIL: actual score ranged from 26 to 97.

c Self-management: actual score scores ranged from 111 to 216.

### Correlation Analysis (Stroke Knowledge, HIL, and Self-Management)

Table 3 shows the correlations between stroke knowledge, HIL, and self-management. Stroke knowledge was positively correlated with self-management (*r*=0.668, *P*<.001) and the HIL score (*r*=0.138, *P*<.001), while HIL was positively correlated with self-management (*r*=0.155, *P*<.001).

**Table 3.** Association between stroke knowledge, health information literacy (HIL), and self-management.

Variable	Self-management	HIL	Stroke knowledge
Self-management	1		
HIL	0.155^a^	1	
Stroke Knowledge	0.668^a^	0.138[Table-fn T3_FN1]^a^	1

a *P*<.001

### The Mediating Effect of HIL (Between Stroke Knowledge and Self-Management)

Based on the mediation effect testing process proposed by Wen and Ye [17], the theoretical model and rationale based on the construction by Tang and Wang [18] and Mao [19], a mediation model was established with self-management as the dependent variable, stroke knowledge as the independent variable, and HIL as the mediating variable. In the first step, regression analysis was performed, where stroke knowledge was the independent variable and self-management was the dependent variable, and the coefficient c was tested. In the second step, regression analysis was performed using stroke knowledge as the independent variable and HIL as the dependent variable, and the coefficient a was tested. In the third step, regression analysis was performed with stroke knowledge and HIL as the independent variables and self-management as the dependent variable, with coefficients b and c. The results showed that coefficients a, b, and c were significant, indicating a partial mediating effect of HIL between stroke knowledge and stroke self-management behaviors. Details are shown in Table 4.

**Table 4.** Mediating effect of health information literacy (HIL) on stroke knowledge and self-management.

Step	Dependent variable	Independent variable	Coefficient	Standardized β coefficient	*t* test^a^ *(df)*	*P* value	Adjusted *R*^2^
1	Self-management	Stroke knowledge	c	1.199	16.87 (1)	<.001	0.356
2	HIL	Stroke knowledge	a	1.957	14.622 (1)	<.001	0.293
3	Self-management	HIL	b	0.277	13.849 (2)	<.001	0.531
		Stroke knowledge	c	0.656	9.084 (2)	<.001	

a The t test was 2-tailed

### Testing the Mediating Effects of HIL

**Figure 2.**
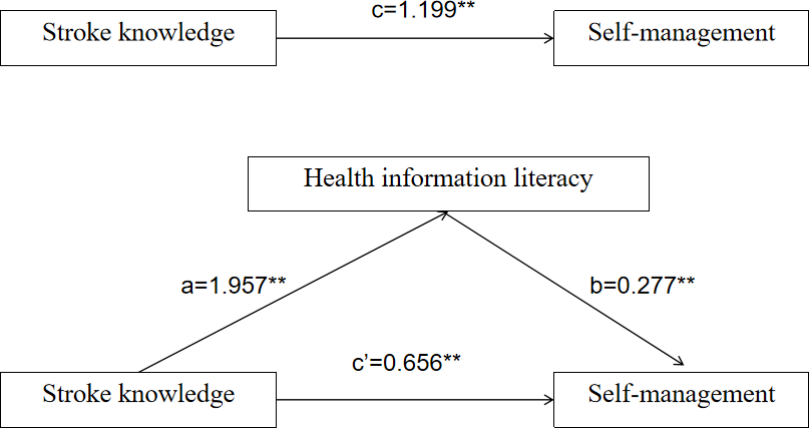
The mediating pathway of health information literacy (HIL) between stroke knowledge and self-management. “a” and “b” are mediating effects of the mediating variable HIL; “c” is a direct effect; **P<.001.

The bootstrap mediation test was conducted using SPSS software (version 26.0). The bootstrap method is a repeated sampling method that treats the original sample as a whole, extracts a large number of new subsamples by repeated sampling with replacement, and obtains statistics. The 95% CI was calculated by repeating the sample 5000 times. The bootstrapped 95% CI for the overall effect of the model did not include a value of 0 (*β*=1.199, 95% CI 1.059, 1.339). The direct effect of stroke knowledge on self-management in terms of bootstrap 95% CI did not include a value of 0 (*β*=.656, 95% CI 0.514, 0.798). The mediated effect path was stroke knowledge, followed by HIL, and finally self-management, and the bootstrap 95% CI for its indirect effect did not include a 0 value (*β*=.543, 95% CI 0.431, 0.663), indicating a significant mediating effect of HIL between stroke knowledge and self-management (Table 5). The mediation pathway is detailed in Figure 2.

**Table 5.** Significance test of the mediating effect of health information literacy (HIL) on stroke knowledge and self-management.

Effect size	Effect	SE	*t* test *(df)*	*P* value	95% confidence limits	
Overall effect	1.199	0.071	*t*=16.870 (512)	＜.001	1.059, 1.339	
Direct effect	0.656	0.072	*t*=9.085 (511)	＜.001	0.514, 0.798	
Indirect effect	0.543	0.058	—^a^	—	0.431, 0.663	

a not applicable.

## Discussion

### Principal Findings and Comparison With Previous Works

There was a positive correlation between stroke knowledge, HIL and self-management. Using the Bootstrap test, the total effect, indirect effect, and direct effect of stroke knowledge on self-management were estimated to be 1.199, 0.543, and 0.656, respectively. The 95% CI did not include 0, indicating the significance of the total effect, indirect effect, and direct effect. This indicates that HIL partially mediates the relationship between stroke knowledge and self-management behavior; stroke knowledge and HIL can directly influence the level of self-management in patients with stroke. Stroke knowledge may also indirectly affect the self-management in patients with stroke through the partial mediating effect of HIL; the direct effect was larger than the indirect effect in this study.

The mean (SD) total knowledge level score of patients with stroke was 17.61(6.457), with the highest score in the dimension of lifestyle, and the lowest score in the knowledge of emergency measures for stroke, which was overall moderate. This result was slightly lower than that reported in other studies [20,21], which was related to factors such as differences in the patients’ educational level and residence in the western region. This study mainly focused on elderly patients with primary and junior high school education, who have relatively weak learning and adaptation abilities [22]. The mean (SD) HIL score of the patients with stroke was 61.17 (13.58), with the highest health information evaluation score and the lowest health information access score among the dimensions, a result consistent with the findings by Wang et al [23]. Although patients with stroke had moderate levels of HIL, they lacked the ability to access high-quality health information. The mean (SD) total self-management score of patients with stroke was 158.7 (19.07) points, which was moderately low, lower than the survey results obtained by Zhou et al [21] but higher than that obtained by Huang et al [24], with the highest score for daily life management and the lowest score for rehabilitation and exercise management among the dimensions. This may be due to a general lack of knowledge about stroke and self-management skills among first-time patients with stroke and their families. Previous studies have shown substantial enhancements in self-efficacy, activities of daily living, and health-related quality of life in patients with stroke following the implementation of self-management interventions [25-27]. Consequently, it is imperative to enhance the self-management competencies of patients with stroke.

A positive correlation was identified between stroke knowledge, HIL, and stroke self-management behaviors. Sufficient knowledge about stroke is a necessary but not sufficient condition for establishing self-management behaviors. In this study, stroke knowledge had a direct effect on self-management, suggesting that the accumulation of more relevant knowledge about stroke disease or treatment and care may directly encourage such patients to adopt healthy coping behaviors and develop good habits. This was also observed by Fisher et al [28], who suggested that information may have a direct impact on behaviors in situations where novel behavioral skills or complex transformation processes are not required in prevention or self-care processes. Self-management is the process of translating professional knowledge guidelines into daily life [29,30]. The ability to obtain health information in HIL directly affects the level of stroke knowledge of an individual. Patients with lower levels of education have restricted access to information due to their limited reading and comprehension skills [18,31]. Health educators can provide more direct knowledge about stroke management through effective health education, such as hospital education, community health lectures, and health manuals. In community education, as shown in the study by Yang et al [32] and Singh et al [33], community-based group rehabilitation and culturally relevant education are effective in improving patients’ quality of life, HIL, and self-management.

HIL is the influencing factor of self-management behaviors. The higher the level of HIL, the more health information is sought, thus promoting more active self-management behaviors [18,22]. However, simple accumulation of knowledge does not necessarily lead to changes in self-management behaviors [34,35], that is, knowledge is not necessarily successfully translated into self-management behaviors. With the COM-B model [36], we can see that the translation of knowledge into behaviors also depends on the ability of patients to recognize the importance of this knowledge. This also depends on their ability to appropriately evaluate the applicability of the knowledge and apply it to health behaviors decisions, and involves a distinction between “knowing knowledge” and “whether to do it.” Stroke self-management is a process that is highly dependent on individual health decision-making, and a weak ability to evaluate information may lead patients to engage in unhealthy behaviors [37,38], in line with the health empowerment theory model. For example, patients who are aware of stroke warning signals may not necessarily respond quickly and take appropriate coping measures, thereby missing the best time for rescue and treatment of the condition. Patients with varying degrees of HIL have unique needs regarding self-management educational content [4,39]. Health professionals must direct their focus towards the older population and those with low literacy levels within their clinical practice, using the HIL scale dimension score to identify patients’ deficiencies and take the individual circumstances of patients into account when formulating a rehabilitation, exercise, and follow-up program as well as self-management education content that is both realistic and patient-centred.

Good HIL means that individuals are aware of the importance of health information and their performance in evaluation and applicability is more pronounced. Good HIL also helps patients to accumulate knowledge about stroke and to make full use of self-management knowledge in health decision-making. As posited by Alzayer et al [40], concerted efforts must be made to enhance awareness and educate the public. These measures ought to be initiated at the outset in order to enhance the HIL level of patients with stroke, facilitate the transformation of knowledge pertaining to stroke into self-management behaviors, and improve the self-management effect of affected patients to ensure optimal treatment outcomes.

### Limitations

Some limitations of this study include the lack of control trials in healthy individuals. Only a population from three hospitals was included, and patients with stroke in the community were not included. The self-report instrument and convenience sampling method may have potential response bias. In addition, this study was a cross-sectional study and could not explore possible causal relationships. Further research should be performed in other regions to confirm and explore intervention studies.

### Conclusions

The self-management of patients with stroke was found to be at a moderate to low level and needs further improvement. There is a correlation between HIL and self-management in various items; the higher the HIL, the better were the self-management behaviors. In addition, HIL is a mediating factor between stroke knowledge and self-management behaviors and can effectively promote the translation of knowledge into behaviors.

## Abbreviations

HIL: health information literacy
