# Correction: Relationship Between Stroke Knowledge, Health Information Literacy, and Health Self- Management Among Patients With Stroke: Multicenter Cross-Sectional Study

**DOI:** 10.2196/80547

**Published:** 2025-07-29

**Authors:** Mengxue Zeng, Yanhua Liu, Ying He, Wenxia Huang

**Affiliations:** 1 Health Management Center, General Practice Medical Center, West China Hospital, Sichuan University, 37 Guoxue Lane, Wuhou District, Chengdu, 610041, China, 86 13980589954; 2 West China School of Nursing, Sichuan University, Chengdu, China

In “Relationship Between Stroke Knowledge, Health Information Literacy, and Health Self- Management Among Patients with Stroke: Multicenter Cross-Sectional Study” (JMIR Med Inform 2025;13:e63956) the authors made one correction.

The affiliations for the first author have been changed from:

*1. West China School of Nursing, Sichuan University, Chengdu, China*

To the following, which has swapped the order of affiliations:

*1. Health Management Center, General Practice Medical Center, West China Hospital, Sichuan University, Chengdu, China*

*2. West China School of Nursing, Sichuan University, Chengdu, China*

The correction will appear in the online version of the paper on the JMIR Publications website, together with the publication of this correction notice, and the numeration of the other author affiliations have been adjusted sequentially. Because this was made after submission to PubMed, PubMed Central, and other full-text repositories, the corrected article has also been resubmitted to those repositories.

